# An Unusual Bacterial Etiology of Fournier’s Gangrene in an Immunocompetent Patient

**DOI:** 10.7759/cureus.26616

**Published:** 2022-07-06

**Authors:** Arshan Khan, Harish Gidda, Nicholas Murphy, Shatha Alshanqeeti, Inderpal Singh, Abdul Wasay, Muhammad Haseeb

**Affiliations:** 1 Internal Medicine, Ascension St. John Hospital, Detroit, USA; 2 Internal Medicine, Wayne State University School of Medicine, Detroit, USA; 3 Internal Medicine, Shalamar Medical and Dental College, Lahore, PAK; 4 Internal Medicine, Bahria International Hospital, Lahore, PAK; 5 Internal Medicine, Jinnah Hospital, Lahore, PAK

**Keywords:** fournier’s gangrene in an immunocompetent patient, fournier's gangrene organisms, fournier's gangrene prognosis and treatment, necrotizing fasciitis, fournier's gangrene (fg)

## Abstract

Fournier's gangrene (FG) is necrotizing fasciitis that affects the penis, scrotum, or perineum. Males are more likely to get affected by this disease. The most common predisposing risk factors are diabetes, alcoholism, hypertension, smoking, and immunosuppressive disorders. FG is a polymicrobial infection caused by both aerobic and anaerobic bacteria. The most common aerobic organisms are *Escherichia coli*, *Klebsiella*, *Proteus*, *Staphylococcus*, and *Streptococcus*. The most common anaerobic organisms are *Bacteroides*, *Clostridium*, and *Peptostreptococcus*. The disease carries high mortality and morbidity, so timely diagnosis and treatment are of utmost importance.

Here, we report a case of a 61-year-old male with a medical history significant for benign prostatic hyperplasia (BPH), who presented to our hospital with fever, watery diarrhea, and painful swelling of the scrotum and penis. The patient was started on piperacillin-tazobactam, vancomycin, and clindamycin. A computed tomography scan of the pelvis showed prostatic enlargement, edema of the penis and scrotum, and air collection within the corpus cavernosum. The patient underwent multiple surgical debridements of the glans penis. Patient wound cultures were positive for *Streptococcus anginosus*, *Actinomyces turicensis*, and *Peptoniphilus harei*. As mentioned earlier, FG is common in diabetic and immunocompromised patients, and infection is usually polymicrobial. Our patient was immunocompetent and his cultures grew atypical organisms.

## Introduction

Fournier’s gangrene (FG) is acute necrotizing fasciitis of the perineum often involving the penis, scrotum, and adjacent tissues [1-3]. It is typically the result of a perirectal or periurethral infection resulting from tissue damage secondary to trauma, medical procedures, or genitourinary disease [1-2]. Common sites of entry include the skin, genitourinary tract, or gastrointestinal tract. FG most commonly affects men with a mean age at presentation of around 50 years. In more than 80% of cases, FG is polymicrobial with common pathogens including *Escherichia coli*, *Klebsiella pneumonia*, *Bacteroides fragilis*, and *Staphylococcus aureus* [4-6]. Here, we present a case of FG with cultures showing *Streptococcus anginosus*, *Actinomyces turicensis*, and *Peptoniphilus harei*. In the literature, there are only a few cases reported of FG caused due to *S. anginosus* and *A. turicensis*. But no case reports were found where *P. harei* was identified as a culprit for FG in an immunocompetent patient.

## Case presentation

A 61-year-old male with a past medical history of chronic bronchitis and benign prostatic hyperplasia (BPH) presented to the hospital after three days of profuse diarrhea, subjective fever, and worsening, painful scrotal and penile swelling. In addition, he noted specks of blood in his urine, dysuria, and mild suprapubic pain. On arrival, he was hypotensive with a blood pressure of 69/46 mmHg, heart rate of 158 beats/minute, respiration rate of 14 breaths/minute, and oxygen saturation of 100%. Physical exam was remarkable for diffuse penile swelling of the glans and shaft, scrotal swelling, blistering, and a 1-cm superficial skin tear on the penis. Testing for chlamydia, gonorrhea, HIV, and trichomonas was negative. The laboratory workup is summarized in Table 1.

**Table 1:** Initial laboratory workup on admission

Test	Results	Reference range
White blood cell count	18.21	5.00-11.00 x 10^3^/uL
Platelet	40	150-400 x 10^3^/uL
Creatinine	4.35	0.5-1.1 mg/dL
Blood urea nitrogen	76	6-23 mg/dL
Brain natriuretic peptide	3887	<101 pg/mL
Anion gap	20	4-14 mmol/L
Lactic acid	3.7	0.5-2 mmol/L
Aspartate aminotransferase	39	1-35 U/L
Alanine aminotransferase	29	1-45 U/L
Alkaline phosphatase	262	38-126 U/L
Bilirubin direct	0.5	0.0-0.8 mg/dL
Bilirubin total	1.3	0.1-1.2 mg/dL
D-dimer	>20,000	<500 ng/mL
Fibrinogen level	539	150-470 mg/dL
Fibrin split products	0.37	<5 ug/mL

**Figure 1:**
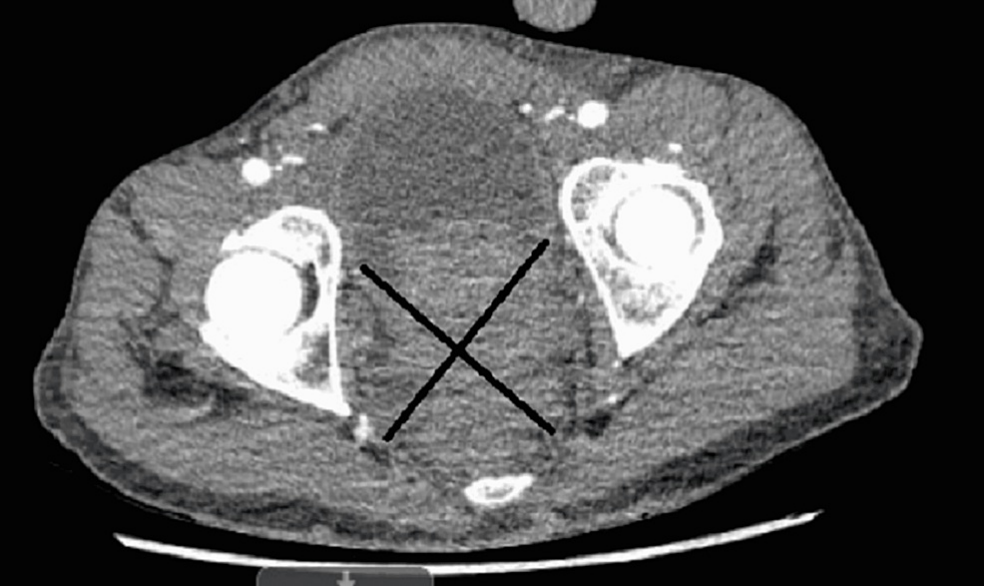
A computed tomography scan of the pelvis revealed prostatic enlargement with a distended bladder

An electrocardiogram (ECG) showed atrial fibrillation with the rapid ventricular response (RVR) that was treated with intravenous (IV) diltiazem. History and physical examination were concerning for FG and the patient was administered broad-spectrum antibiotics including piperacillin-tazobactam 4500 mg every eight hours, vancomycin with a starting loading dose of 25 mg/kg, and clindamycin 600 mg every eight hours. A computed tomography (CT) scan revealed prostatic enlargement with a distended bladder and penile and scrotal soft tissue edema (Figure 1). There were also small air collections within the corpus cavernosum. He had difficulty urinating with subsequent foley catheter placement that drained around 1.3 L. Scrotal ultrasound was significant only for diffuse scrotal wall thickening and edema. Penile duplex ultrasound also noted possible foci of air within the corpus spongiosum. Cavernosal artery blood flow was without obstruction. Two sets of blood cultures grew *Haemophilus haemolyticus* and *A. turicensis*.

Two days after arrival, he was vitally stable and his presenting symptoms were improving except for the genital swelling. Further examination revealed new crepitus of the distal glans penis. His white blood cell (WBC) count remained elevated at 18,000 cell/mcL and platelets were as low as 34,000 cell/mcL, necessitating platelet transfusion for urgent surgical exploration. The glans penis was incised and noted to be necrotic in appearance. Dark red blood was drained without purulence or odor with similar findings on incision of the proximal penis. Cultures were taken during the procedure which grew *S. anginosus*, *A. turicensis*, and *P. harei*. Antibiotic treatment was changed to ampicillin-sulbactam 3000 mg every six hours after the bacterial sensitivity came back.

**Figure 2:**
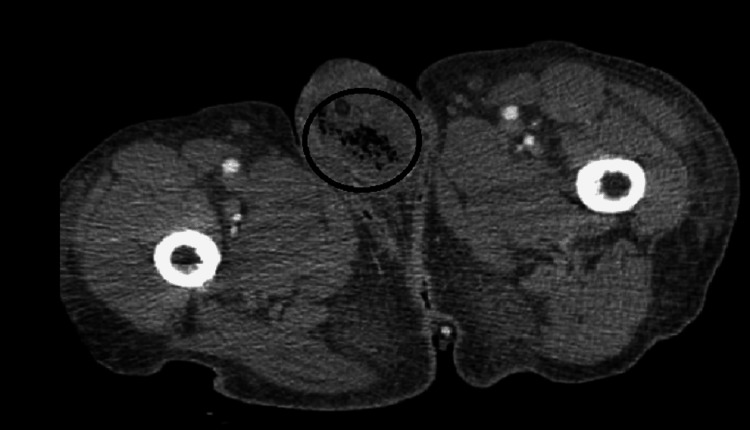
A computed tomography scan of the abdomen and pelvis on day 5 of admission revealed gas in the anterior penis

Five days later, his WBC count increased to 34,000 cell/mcL and CT of the abdomen/pelvis showed increased soft tissue gas in the anterior penis (Figure 2). The next day, he returned to the operating room with urology for debridement of the glans. During debridement, a suprapubic catheter was placed and cystoscopy revealed the first 6 cm of the urethra to have a moth-eaten appearance with another similar segment around 1 cm long near the bulbar urethra. Tissue samples from the glans showed extensive necrosis and acute inflammation noted as clinical gangrene.

After the second debridement, the ampicillin/sulbactam combination was changed to amoxicillin/clavulanate 875/125 mg twice daily. Leukocytosis resolved and D-dimer declined to 5000 ng/mL. A week after the second debridement, the tissue was again sharply dissected off the ventral glans down to viable tissue, done at the bedside. There was no crepitus and the wound edges appeared granulated and healing.

He was clinically stable and improving at 18 days post-admission when he was discharged. Amoxicillin/clavulanate was to be continued outpatient for a total combined length of 21 days of antibiotic therapy.

## Discussion

FG is a devastating infection involving the perineum, scrotum, and penis in men and labia in women. Men are more commonly affected than women [7]. Its annual incidence is estimated to be 500-1000 cases. Necrotizing fasciitis affects 0.40 cases per 100,000 in the United States, and up to 1 in every 100,000 in other parts of the world. The disease often occurs from infections of the urogenital tract, anorectal area, or skin of the genitals. Other common risk factors for FG development are immunocompromised patients, diabetes, obesity, and cancers [4].

Patients often have an inciting event such as skin or mucosal tear and decreased integrity. Some examples are penetrating trauma, urological/gynecological/analogical procedures, insect bites, skin tears, and hemorrhoids. Infections are often polymicrobial with anaerobic and aerobic bacteria infecting the fascial planes [8]. Common organisms grown in wound cultures are Group A *Streptococci*, *S. aureus*, and gram-negative such as *E. coli* and *Pseudomonas* [7-8].

Early detection and diagnosis are critical as it can rapidly progress and even lead to death [9]. Findings suggesting necrotizing fasciitis include erythema, edema, systemic signs of infection, edematous, dull gray, questionable exudate, tissue separated easily by blunt dissection, and crepitus [2,9]. Additional skin findings of bullae or necrosis may be evident. Patients may also have severe pain out of proportion to examination findings. Blood cultures and wound cultures should be obtained. Finding the organism(s) and susceptibilities can aid antibiotic regimens. Elevated aspartate aminotransferase (AST) and creatine phosphokinase (CPK) suggest muscle involvement; leukocytosis with left shift is commonly seen in FG. Imaging may aid in the diagnosis, especially when crepitus is observed; a CT scan is the best initial radiographic imaging and may show the presence of gas in the tissue. Surgery should not be delayed for imaging. Surgical exploration is the only way to establish the diagnosis [9-10]. Intraoperative wound cultures should be obtained.

The cornerstones of treatment of FG are intensive hemodynamic support with aggressive fluid resuscitation, emergency surgical debridement, and broad-spectrum antibiotics [1-2]. FG should be considered a surgical emergency, and urgent surgical debridement is critical to ensure a successful outcome. All necrotic tissues should be debrided and excised [3-5]. Delay in surgical debridement is associated with high mortality and morbidity. In a retrospective study of 72 patients with FG, a delay in surgical debridement was associated with a significant increase in mortality [6]. Repeated surgical debridement is necessary in the case of infection progression or extensive necrotic tissue [7].

Most cases of FG are polymicrobial; therefore, the broad-spectrum antibiotics should be started immediately after obtaining blood cultures and, if possible, tissue cultures. The antibiotic regimen should cover *Staphylococcus*, *Enterococcus*, *E. coli*, and other gram-negative pathogens and anaerobes (including *Bacteroides *and *Clostridium* species) [8]. The current Infectious Diseases Society of America (IDSA) guidelines recommend vancomycin or linezolid plus one of the following agents: piperacillin-tazobactam, a carbapenem, or ceftriaxone-metronidazole. Antibiotics should be tailored to Gram stain, culture, and sensitivity results when available [9-10]. The use of protein synthesis inhibitors, such as clindamycin, may help by inhibiting toxin production, particularly in those with clostridial and streptococcal infections [10]. Intravenous immune globulin (IVIG) and clindamycin should be used for patients with streptococcal toxic shock syndrome (TSS). The use of IVIG is supported by a meta-analysis where the use of IVIG in patients with streptococcal TSS decreased mortality from 33.7% to 15.7% [11]. Antimicrobial administration should be continued until no further debridement is needed, and the patient improves clinically [9].

Negative pressure wound therapy and wound vacuum-assisted closure (VAC) is commonly used after surgical debridement. Wound VAC therapy increases the blood flow to the affected tissue and helps to clear the infection [12]. Hyperbaric oxygen is sometimes used as adjuvant therapy to the affected tissue to prevent the growth of anaerobic bacteria, but its use is controversial [13].

Necrotizing skin infections are associated with very high mortality, even with appropriate medical therapy. The mortality rate for FG is 22%-24% [14]. The mortality can be reduced by early diagnosis, early initiation of antibiotics, and urgent surgical debridement.

## Conclusions

FG has a high mortality, and it is primarily seen in immunocompromised patients, but it can also be seen in immunocompetent patients, as in our case. The clinician should have a high index of suspicion of FG even when an immunocompetent patient presents with penis, scrotum, or perineal swelling and pain out of proportion to the physical findings. The key to treatment is urgent surgical debridement, intravenous antibiotic, and aggressive intravenous fluid resuscitation. Infection is polymicrobial, and the initial antibiotic regimen should cover *Staphylococcus*, *Enterococcus*, *E. coli*, and other gram-negative pathogens and anaerobes (including *Bacteroides *and *Clostridium* species) as they are the most common culprits. Sometimes FG can be caused by an atypical pathogen, as in our case. Antibiotics should be tailored according to Gram stain and cultures afterward.
